# Treatment of Typhoid Fever

**Published:** 1878-10-20

**Authors:** 


### TREATMENT OF TYPHOID FEVER.

In a lecture by Dr. Alonzo Clark, reported in the N. Y. *Med. Record*, he remarks: I may safely say to you that a case of typhoid fever of average severity needs no medicine except for the relief of certain symptoms, such as sleeplessness, perhaps a little urgency in the diarrhœa, sensation of burning on the surface of the body, etc. There are a great many cases of typhoid fever which need no treatment whatever by way of drugs, but everything by way of management of the case. Still, it does happen in many of these cases that some one of the symptoms requires treatment. The diarrhœa, for example, in many cases requires restraint.

Diarrhœa does not occur in every case of typhoid fever in this country ; perhaps it does not occur in two-thirds of the cases. The astringent I have referred to so frequently is found to answer a very good purpose. It consists of:

℞. Bismuth. subnit....dr.i.
Morphiæ sulph....gr. i.
M. et. div. in chart. No. xii.
One to four a day.

The common astringents tr. kino and tr. catechu may be employed, and the decoction of blackberry root is sometimes very servicable. In some cases it requires the moderate free use of opium to restrain the diarrhœa.

There is always a *cough* in typhoid fever, but as it is not important in the average case, I have not mentioned it until now. There is slight bronchial irritation, which appears early in the disease, and continues usually until the period of imperfect anæsthesia is reached, then it may cease. The material raised is commonly a glairy mucus, but in some cases the slight bronchitis becomes a catarrh, and will require treatment. It will need the same treatment as bronchitis occurring under any other circumstances, except that the tonic expectorants will be most likely to do good. Perhaps one of the best that can be used is the *Co. Tr. of Benzoin*, in doses of ten drops on sugar once in three or four hours. A very good combination is the tincture of the balsam of tolu and the mistura guaiaci.

℞. Mist. guaiaci....dr. j. to ℥ss.
Tr. balsam tolu.....gtts. vj. to x.
M.

This can be repeated every two, three, or four hours. Sometimes the inhalation of the vapors of warm water seems to be required for one or two hours each day.

*Restlessnezs* is one of the prominent features of the disease, and that will very frequently be entirely quieted by sponging the surface of the body with warm or cold water. If the temperature is high cold water is better than warm ; and in some cases a Dover powder will be required.

The *temperature of the body* will require your attention. In many cases of typhoid fever it does not rise to a dangerous point; in a few cases it does. You will see the greater number of cases go through the entire course of the disease without the temperature at any time reaching 105° F. In a case of average severiety the maximum temperature is about 104$_{\circ}$ F. in occasional cases it reaches 106° F. or 107° F., and then you will either give quinine in pretty decided doses or use cold water for its reduction. If the patient is a young person, the cold bath is the most convenient means of reducing the temperature, and certainly the most efficacious. The temperature of the bath should be only ten degrees below the temperature of the body when the patient is first put into it. If the temperature of the body be 102° F., the patient may be placed in a bath having a temperature of 95° F.; then some of the warm water can be removed, and be replaced by cold water until the bath has been reduced to 80° F. If the patient is permitted to remain in the bath twenty minutes, the temperature is usually reduced 1 2 3 4 or 5 degrees.

He is then removed from the bath, put back into bed, and it will be ssveral hours, usually, before the temperature will rise as high as it was before using the bath. When it rises, another bath is to be given, and in that manner you will go on repeating the bath as often as may be necessary to keep the temperature below the point of danger.

The son of one of the Professors in the college has within the present season had typhoid fever. In his case the bath was used about five times a day for several days, and always with the result of reducing the temperature and affording great relief to the patient.

For the hemorrhage from the bowels there is but little that can be done, unless, in addition to absolute rest, the fluid extract of ergot be administered.

For the perforation of the bowels, I have some faith in the opium treatment. As I told you, I feel confident that I saved one doctor's life by the narcotizing influence of opium, and there is no objection in typhoid fever to the administration of this drug.

"Grave fed fevers," typhoid fever as well as typhus; and now we come to the two essentials in the treatment of this disease. I am in the habit of repeating the old proverb, "Stuff a cold, and starve a fever," and then add that we stuff them both now. First, then, the administration, steadily and preservingly, of such food as can be absorbed by the stomach. We cannot talk much of digestion; the stomach is in a diseased condition, and cannot digest well, consequently everything solid in the way of food is out of the question. Most of these patients dispose of milk pretty well. For all those who can dispose of it, milk is the best food that can be used. For those who cannot use it, you will be obliged to do the best you can with beef-tea, raw egg beaten up with water, and made of such consistancy that it can be taken with a spoon; and the expressed juice of beef. The beef-tea does not contain a great deal of nourishment, and when it can be used, milk is a much better article of food.

The expressed juice of beef answers very well, and can be obtained by cooking a piece of steak so as just to crust the two surfaces, and then cutting it into pieces and squeezing the juice out with a lemon-squeezer. The broths are given rather as diluted food in the early part of the disease, when it is supposed that the patient should not take much nourishment, but as the disease advances, the food should be more and more sustaining. In cases in which the stomach fails to retain the food, nutritious enemata should be employed. You will remember that the disease which produces the diarrhœa, is in the small intestine, not in the large.

The other essential of which I wish to speak is fresh air, but I wiil reserve that for the opening of the next lecture.

